# Bacteriophage and antibiotic combination therapy for recurrent *Enterococcus faecium* bacteremia

**DOI:** 10.1128/mbio.03396-23

**Published:** 2024-02-14

**Authors:** Madison E. Stellfox, Carolyn Fernandes, Ryan K. Shields, Ghady Haidar, Kailey Hughes Kramer, Emily Dembinski, Mihnea R. Mangalea, Garima Arya, Gregory S. Canfield, Breck A. Duerkop, Daria Van Tyne

**Affiliations:** 1 Department of Medicine, Division of Infectious Diseases, University of Pittsburgh, Pittsburgh, Pennsylvania, USA; 2 Department of Immunology and Microbiology, University of Colorado School of Medicine, Aurora, Colorado, USA; Emory University, Atlanta, Georgia, USA; University of California, San Diego, San Diego, California, USA

**Keywords:** vancomycin-resistant *Enterococcus faecium*, bacteriophage therapy, phage-neutralizing antibodies

## Abstract

*Enterococcus faecium* is a member of the human gastrointestinal (GI) microbiota but can also cause invasive infections, especially in immunocompromised hosts. Enterococci display intrinsic resistance to many antibiotics, and most clinical *E. faecium* isolates have acquired vancomycin resistance, leaving clinicians with a limited repertoire of effective antibiotics. As such, vancomycin-resistant *E. faecium* (VREfm) has become an increasingly difficult to treat nosocomial pathogen that is often associated with treatment failure and recurrent infections. We followed a patient with recurrent *E. faecium* bloodstream infections (BSIs) of increasing severity, which ultimately became unresponsive to antibiotic combination therapy over the course of 7 years. Whole-genome sequencing (WGS) showed that the patient was colonized with closely related *E. faecium* strains for at least 2 years and that invasive isolates likely emerged from a large *E. faecium* population in the patient’s gastrointestinal (GI) tract. The addition of bacteriophage (phage) therapy to the patient’s antimicrobial regimen was associated with several months of clinical improvement and reduced intestinal burden of VRE and *E. faecium. In vitro* analysis showed that antibiotic and phage combination therapy improved bacterial growth suppression compared to therapy with either alone. Eventual *E. faecium* BSI recurrence was not associated with the development of antibiotic or phage resistance in post-treatment isolates. However, an anti-phage-neutralizing antibody response occurred that coincided with an increased relative abundance of VRE in the GI tract, both of which may have contributed to clinical failure. Taken together, these findings highlight the potential utility and limitations of phage therapy to treat antibiotic-resistant enterococcal infections.

**IMPORTANCE:**

Phage therapy is an emerging therapeutic approach for treating bacterial infections that do not respond to traditional antibiotics. The addition of phage therapy to systemic antibiotics to treat a patient with recurrent *E. faecium* infections that were non-responsive to antibiotics alone resulted in fewer hospitalizations and improved the patient's quality of life. Combination phage and antibiotic therapy reduced *E. faecium* and VRE abundance in the patient's stool. Eventually, an anti-phage antibody response emerged that was able to neutralize phage activity, which may have limited clinical efficacy. This study demonstrates the potential of phages as an additional option in the antimicrobial toolbox for treating invasive enterococcal infections and highlights the need for further investigation to ensure phage therapy can be deployed for maximum clinical benefit.

## OBSERVATION

Enterococci are hardy and adaptable gastrointestinal (GI) tract commensal organisms found in almost all terrestrial animals (1). Enterococci display intrinsic resistance to many antibiotics, and most clinical *Enterococcus faecium* isolates in the United States have acquired vancomycin resistance, leaving clinicians with a limited repertoire of effective antibiotics. These characteristics have allowed enterococci to become successful nosocomial pathogens, especially in immunocompromised populations (2). Bacteriophages (phages) offer a potential adjunctive tool to manage infections with multi-drug-resistant bacteria (3, 4). However, few reports have demonstrated *in vivo* efficacy of phage therapy for *E. faecium* infections in humans (5, 6). Several knowledge gaps remain regarding the effects of phage therapy on GI tract microbiota and whether host immune responses complicate phage therapy. Here, we present an in-depth characterization of the clinical, microbiological, and host immune response aspects of phage therapy in a patient with several years of recurrent *E. faecium* bacteremia.

**Fig 1:**
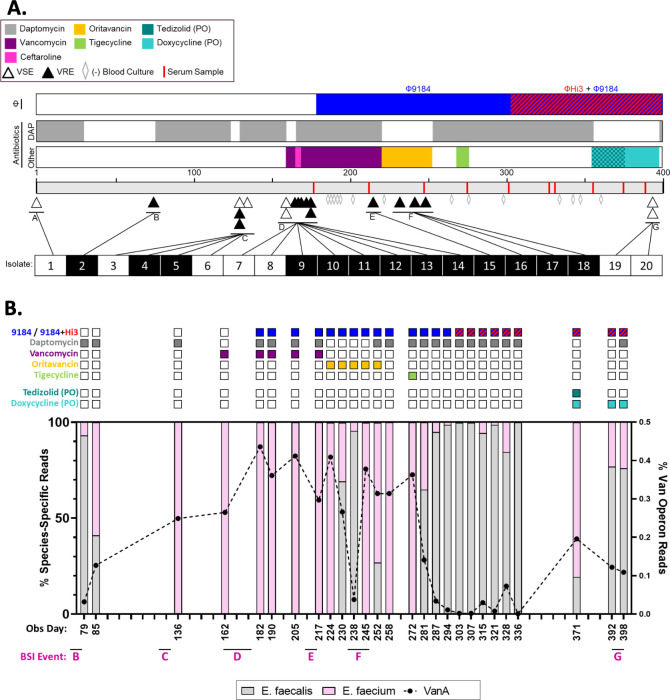
Clinical timeline and GI tract enterococcal populations. (**A**) The timeline represents the observation period (June 2020 through July 2021). The three bars above the timeline depict the type and duration of treatments used throughout the observation period: blue (Φ9184) and red (ΦHi3) for phages, gray for daptomycin exposure, and the multicolored bar highlights other systemic antibiotics given (vancomycin, purple; ceftaroline, pink; oritavancin, yellow; tigecycline, green; tedizolid, dark teal; doxycycline, light teal). PO indicates that the antibiotic was given orally, and all other antibiotics were given intravenously. Triangles below the timeline indicate positive blood cultures that grew vancomycin-sensitive *E. faecium* (VSE, open triangles) and vancomycin-resistant *E. faecium* (VRE, closed triangles). Gray, open diamonds indicate blood cultures that were obtained after starting phage therapy but remained negative. Red dashes indicated time points when serum was collected to test for phage neutralization. Below the timeline, BSI isolates that underwent WGS are numbered 1 through 20 and are shaded white for VSE and black for VRE. (**B**) GI enterococcal population metagenomics of pooled colonies from stool samples plated on bile esculin azide agar. The observation day indicates when each stool sample was collected. The relation to BSI events is listed below and corresponds to BSI events described in panel A. The bar graph shows the relative abundance of reads mapping to *E. faecalis* (gray bars), *E. faecium* (pink bars), and the VanA operon (black dotted line). Colored squares above each bar indicate which phage (top row), systemic intravenous antibiotics (rows 2–5), and oral antibiotics (rows 6 and 7) the patient was being treated with at the time each stool sample was collected.

The patient was a 57-year-old female with a past medical history significant for prior Roux-en-Y bariatric surgery, recurrent extended-spectrum beta-lactamase (ESBL)-producing Gram-negative urinary tract and pulmonary infections, and mild immunosuppression secondary to treatment for Sjogren’s syndrome and adrenal insufficiency. She was known to our medical center, UPMC, for recurrent *E. faecium* bloodstream infections (BSIs) beginning in 2013. She underwent several hospitalizations for *E. faecium* BSI between 2013 and 2020 and received multiple courses of therapeutic and suppressive antibiotics. No focal source of infection was discovered despite extensive diagnostic imaging, including multiple transthoracic and transesophageal echocardiography examinations, tagged white blood cell and PET/CT scans, as well as endoscopy and colonoscopy procedures. Therefore, the patient’s recurrent bacteremia was attributed to reseeding from her GI tract microbiota. Starting in June 2020 (observation day 1), the patient experienced increased frequency and duration of *E. faecium* BSI events (Fig. 1A). This culminated in 26 days of persistent *E. faecium* bacteremia despite treatment with multiple antibiotics displaying *in vitro* activity (Event D, Fig. 1A). The patient was subsequently referred for salvage phage therapy.

A previously characterized siphovirus phage, Φ9184 (7), had lytic activity against clinical isolates collected from the patient. After obtaining emergency investigational new drug (eIND) approval by the FDA, local IRB approval, and informed patient consent, Φ9184 was added to systemic antibiotics on observation day 182 at 1 × 10^9^ plaque forming units (PFU) per dose. The phage formulation was given three times daily both intravenously (IV) and orally (PO) in an effort to target the suspected source of the recurrent infections, the GI tract. To prevent degradation of the phage in the stomach, the patient was maintained on a proton pump inhibitor regimen. After 26 days of persistent infection while on daptomycin (which targets the bacterial cell membrane and causes rapid depolarization), in combination with either vancomycin or ceftaroline, which both target different components of the bacterial cell wall (by binding to D-alanyl-D-alanine and penicillin-binding proteins, respectively), bacteremia resolved within 24 hours of phage administration, and the patient was discharged home soon afterward on both antibiotic therapy (which alone had failed to eradicate the infection) and phage therapy (Fig. 1A).

While receiving concurrent treatment with daptomycin, vancomycin, and Φ9184, a single outpatient blood culture was positive for VREfm on day 213 (Event E, Fig. 1A). Antibiotic therapy was switched from daptomycin and vancomycin to weekly oritavancin infusions, another cell wall targeting antibiotic, but bacteremia again recurred (Event F, Fig. 1A). A subsequent trial of daptomycin in combination with tigecycline, which inhibits bacterial ribosomal activity and therefore protein synthesis, was not tolerated due to tigecycline-related GI side effects, and the patient was continued on daptomycin and Φ9184 alone. During this time, all breakthrough BSIs were able to be managed in the outpatient setting in accordance with patient and family preference. However, these BSI recurrences prompted a search for additional phages.

On day 303, ΦHi3 (related to a previously characterized siphovirus phage, Φ9183 (7)) was added to the treatment regimen alongside Φ9184 for a total of 2 × 10^9^ PFU/dose. This doubled the previous Φ9184 monotherapy dose. The patient remained bacteremia-free for 4 months, was able to travel, and was hospitalized only once in the 6 months after starting phage therapy for a non-BSI-related reason. IV daptomycin was eventually discontinued after an extended 14-week treatment course, and the patient was switched to oral suppressive therapy with doxycycline and tedizolid (both ribosomal-targeting and protein synthesis-inhibiting antibiotics). The PO and IV phage regimen was also decreased to twice daily maintenance therapy, to simplify administration at home. However, on day 395, the patient developed a recurrent *E. faecium* BSI (Event G, Fig. 1A).

### Bacterial genomics and stool metagenomics

Isolates from every BSI event as well as *E. faecium* from a stool sample (collected on day 80) and historical epidemiologic surveillance rectal swabs (collected 18 and 6 months prior to the first day of observation) underwent WGS on the Illumina platform. A patient-specific, closed reference genome was generated with long-read sequencing and hybrid assembly for the earliest isolate (supplemental methods). Genomic analysis indicated that all BSI isolates were closely related to isolates from the GI tract, in support of our hypothesis that the source of infection was the patient’s GI enterococcal population (Fig. S1). To better understand how phage therapy affected the GI enterococcal population, we performed shotgun metagenomics on 100–1,000 pooled colonies from stool samples collected routinely throughout the observation period that were plated on enterococcal-selective media (supplemental methods). Prior to initiation of phage therapy, the proportion of reads mapping to the *E. faecium* genome and to the VanA operon steadily increased over time and remained dominant despite significant exposure to daptomycin, vancomycin, as well as Φ9184 (Fig. 1B). Treatment with oritavancin, a cell-wall active glycopeptide antibiotic to which the patient was naïve, was associated with a transient decrease in *E. faecium* and VanA operon abundance, but a subsequent increase in *E. faecium* burden was temporally related to the breakthrough BSI event F (Fig. 1B). However, after a brief period of tigecycline exposure and the addition of ΦHi3 to Φ9184, both the *E. faecium* and VanA operon abundance in the GI tract again decreased and remained suppressed for several months. During this time (days 248–394), there were no BSI episodes detected despite surveillance blood cultures (Fig. 1A, gray diamonds). We also performed 16S rRNA sequencing on the same stool samples shown in Fig. 1B, and the relative abundance of enterococci was similarly decreased while the patient was receiving Φ9184 and oritavancin and while she was receiving the two-phage cocktail and daptomycin (Fig. S2). These data suggest that neither systemic antibiotics alone nor the combination of systemic antibiotics and Φ9184 durably altered the enterococcal population in the GI tract. However, the administration of tigecycline and/or the addition of ΦHi3 to the phage cocktail suppressed the *E. faecium* population in the GI tract and prevented BSI recurrence for 147 days.

### Phage–antibiotic susceptibility testing

**Fig 2:**
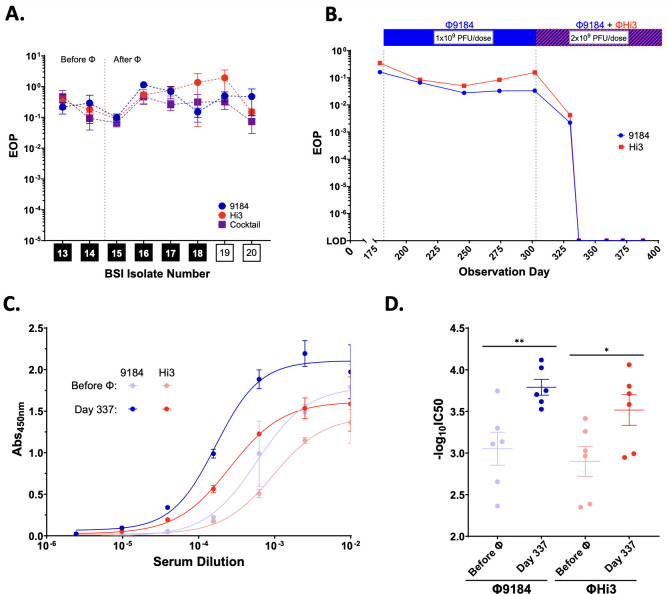
Development of a phage-specific neutralizing immune response. (**A**) Efficiency of plating (EOP) of Φ9184 (blue circles), ΦHi3 (red circles), and a 50:50 cocktail of both phages (purple squares) on clinical *E. faecium* BSI isolates compared to the phage host strain. Isolates 13 and 14 were the last isolates collected prior to phage initiation (Before Φ), and isolates 15–20 represent breakthrough BSI events occurring after phage therapy was started (After Φ). Black boxes indicate VRE, and white boxes indicate VSE isolates. (**B**) Average EOP of Φ9184 and ΦHi3 on the host strain after incubation with patient serum versus incubation with buffer alone, across three replicates. (**C**) ELISA IgG-binding curves from host sera against Φ9184 (blue) and ΦHi3 (red). “Before Φ” indicates the serum sample that was collected prior to initiation of phage therapy (observation day 181), and "Day 337” indicates the serum sample collected on observation day 337, which was 34 days after starting the Φ9184 and ΦHi3 cocktail. (**D**) Half-maximal IgG titers (-log_10_ EC50) of each ELISA replicate from the conditions shown in panel C. Error bars indicate the standard error mean of replicates. *P < 0.05 and **P < 0.01.

Despite clinical improvement, the patient had a BSI recurrence on day 395. Throughout treatment, we evaluated all bacterial isolates collected during breakthrough BSI events for the emergence of antibiotic and phage resistance. While we were able to generate phage-resistant mutants to Φ9184 and ΦHi3 *in vitro* (Fig. S3A through C), we observed no appreciable decrease in phage activity as compared to the phage host strain in any of the clinical isolates collected after initiating phage therapy (Fig. 2A). In addition, none of the clinical isolates developed resistance to any of the antibiotics used in combination with phage therapy (Table S1). We also looked for antibiotic–phage antagonism in BSI isolate 20, collected from the final breakthrough BSI event G, but instead observed enhanced bacterial growth suppression when the phage cocktail was used in combination with daptomycin as compared to either phage or daptomycin alone (Fig. S3D). Therefore, it seems unlikely that breakthrough isolates arose due to reduced efficacy of antibiotics, phage, or combination therapy.

### Humoral immunity to phage

As we did not observe phage or antibiotic resistance associated with phage–antibiotic combination failure in this patient, we next assessed for potential immune system interference by monitoring the patient’s serum for evidence of phage neutralization. Human serum can have nonspecific inhibitory effects on phage activity, and we saw a minor (<10 fold) decrease in phage activity when Φ9184 or ΦHi3 was incubated with commercially available pooled human serum (Fig. S4 and supplemental methods) (8, 9). Phage therapy was started on observation day 189, and incubating phages with serum collected on day 179 (before phage exposure) as well as serum collected before day 303 (when the patient was started on the combination of Φ9184 and ΦHi3) resulted in no appreciable decrease in the efficiency of plating (EOP) for either phage below the expected nonspecific inhibition of human serum (Fig. 2B). However, serum collected 27 days after starting ΦHi3 (day 330) caused an almost 100-fold reduction in the EOP for both phages, and all subsequent serum samples completely neutralized phage activity to below the limit of detection (EOP <1×10^−7^) (Fig. 2B).

To further characterize the anti-phage component of the serum neutralization response, we quantified IgG binding to ultra-purified Φ9184 or ΦHi3 using a custom ELISA assay (supplemental methods). Serum collected after complete neutralization was detected (day 337) showing a significant increase in the half-maximal IgG titers against both Φ9184 and ΦHi3 as compared to serum collected prior to phage therapy (day 181) (Fig. 2C and D). These data suggest that switching from phage monotherapy (Φ9184) to a 2-phage cocktail (adding ΦHi3 to Φ9184) and/or doubling the dose triggered a neutralizing antibody response that may have inhibited phage activity in the bloodstream and could have contributed to the final breakthrough BSI (event G). As such, after 6 months of treatment, phage therapy was discontinued, and the patient and her family decided to transition to hospice care. The patient continued to experience intermittent *E. faecium* bacteremia until she passed away due to pneumonia 7.5 months after discontinuation of phage therapy.

### Discussion

*E. faecium* displays resistance to many first-line antibiotics and is regularly associated with persistent and recurrent infections. In this study, systemic antibiotics alone were unable to achieve durable remission of recurrent *E. faecium* BSIs in this patient. A phage cocktail combined with traditional antibiotics temporarily suppressed recurrent BSIs and reduced intestinal *E. faecium* burden, which resulted in fewer hospitalizations and improved quality of life for 6 months. These observations highlight the clinical relevance of prior *in vitro* and pre-clinical studies detailing the synergistic effects of phage cocktails and antibiotic combinations on enterococcal infections (7, 10–14). While this study investigated several crucial aspects of phage therapy with the intent to inform the medical and scientific community, it consisted of data acquired during clinical care of a single patient. Our observations are suggestive of the tolerability, benefits, and pitfalls of phage therapy for *E. faecium* infections, but without further study and well-designed clinical trials, these results may not be generalizable to a larger patient population. Nevertheless, currently, there is sparse literature describing clinical experience with *E. faecium-*targeting phages, and few studies have assessed phage-associated changes to human gut microbial communities. Additionally, host immune responses are increasingly recognized as a potential limitation of phage therapy (15, 16). To harness the full potential of phages in treating difficult enterococcal infections, future studies should continue to monitor treatment-related microbiome changes and host immune responses to maximize the clinical efficacy of this technology.

## SUPPLEMENTAL MATERIAL

Supplemental material mbio.03396-23-s0001.docx Supplemental methods and Figures S1-S4.

Table S1 mbio.03396-23-s0002.xlsx Clinical microbiological data for all *E. faecium* isolates collected in the study.

ASM does not own the copyrights to Supplemental Material that may be linked to, or accessed through, an article. The authors have granted ASM a non-exclusive, world-wide license to publish the Supplemental Material files. Please contact the corresponding author directly for reuse.
