# Supplementary material for: Bacteriophage and antibiotic combination therapy for recurrent Enterococcus faecium bacteremia
Source: mBio. 2024 Feb 14;15(3):e03396-23. doi: 10.1128/mbio.03396-23 (PMC10936196; doi:10.1128/mbio.03396-23)
Supplement: Supplemental material — Supplemental methods and Figures S1-S4. [file mbio.03396-23-s0001.docx]

**SUPPLEMENTAL MATERIAL**

**Supplemental Methods**

Study design

This was a prospective observational case study of a single patient with recurrent *E. faecium* bacteremia that received bacteriophage (phage) therapy. The patient was referred to our laboratory at the request of the attending infectious disease physician and informed patient consent was obtained. FDA emergency investigational new drug approval for administration of phages was obtained (eIND #27183), and the local Institutional Review Board also approved the study (Protocol #EA20120145). The first day of patient observation was in June 2020 and extended through July 2021. Invasive BSI isolates from positive blood cultures were obtained through the UPMC clinical microbiological laboratory. Standard antibiotic minimum inhibitory concentrations (MICs) were measured on the MicroScan WalkAway System (Beckman Coulter), while additional susceptibility testing for tigecycline, telavancin and quinupristin/dalfopristin was tested by E-test (Supplemental Table 1). Throughout the follow-up period, weekly collection of whole blood and stool samples were planned but were not always available due to outpatient care logistics.

*E. faecium* isolate whole genome sequencing and assembly

Two VRE isolates from rectal swabs collected from the patient 18- and 6-months prior to the first day of observation (isolates labeled REF and PRE, respectively in Supplemental Table 1 and Fig. S1) were available from hospital VRE surveillance efforts that were ongoing during this study. We also collected three clones of *E. faecium* from a stool sample collected from the patient on day 80. Additionally, 20 *E. faecium* isolates collected from BSI events (A – G) were obtained with assistance from the clinical microbiological laboratory.

A total of 25 isolates underwent whole genome sequencing (WGS) on the Illumina platform. Genomic DNA was extracted with a Qiagen DNeasy Blood and Tissue Kit (Qiagen, Hilden, Germany) and Illumina sequencing libraries were made with a Nextera kit (Illumina, San Diego, CA). Libraries were sequenced on a NextSeq 500 using 2x150-bp paired-end reads. The REF (t-18 month) isolate was also sequenced on the Oxford Nanopore Technologies MinION platform, and hybrid assembly was used to generate a patient-specific reference genome with Unicycler [(17)](https://sciwheel.com/work/citation?ids=3964715&pre=&suf=&sa=0&dbf=0). Genomes were analyzed using tools available through the Center for Genomic Epidemiology (<http://www.genomicepidemiology.org/>), and snippy (https://github.com/tseemann/snippy). All isolates were found to belong to multi-locus sequence type (ST) 17 and were separated from the patient-specific reference genome by 18 – 61 single nucleotide polymorphisms (SNPs). A SNP-based, core genome phylogenetic tree was generated with snippy and constructed with RAxML [(18)](https://sciwheel.com/work/citation?ids=265260&pre=&suf=&sa=0&dbf=0) with 100 bootstraps and visualized with iTOL (https://itol.embl.de/login.cgi). The estimated SNP accumulation rate was approximately 18 SNPs/year, which is within previously published ranges for *E. faecium* [(19, 20)](https://sciwheel.com/work/citation?ids=3974047,5151535&pre=&pre=&suf=&suf=&sa=0,0&dbf=0&dbf=0).

Enterococcal metagenomics

Stool samples were plated onto bile esculin azide agar (BD 212205, Fisher Scientific) and incubated overnight at 37°C. 100 – 1000 colonies were pooled, and genomic DNA was extracted and sequenced on the Illumina platform. Raw reads were assessed for quality (minimum phred quality of 30) and adapters were trimmed with fastp [(21)](https://sciwheel.com/work/citation?ids=5861897&pre=&suf=&sa=0&dbf=0). Reads with >90% identity were mapped to the VanA operon with bbmap and the percentage of total reads was calculated (https://sourceforge.net/projects/bbmap/). To determine the species distribution of the pooled enterococcal colonies, a custom database of 1337 enterococcal genomes was constructed from the NCBI GenBank database including all assemblies for Taxon ID 1350 (*Enterococcus)* with a Complete Genome assembly level (accessed April 2021). Reads were cleaned as above and all metagenome samples were species classified using Kraken 2 with a confidence score of 0.9 [(22)](https://sciwheel.com/work/citation?ids=8120680&pre=&suf=&sa=0&dbf=0). The relative abundance was calculated of the two predominant species, *E. faecalis* and *E. faecium*.

Stool 16S metagenomics

Aliquots of the same stool samples as analyzed above had been stored at -80°C in SM+ buffer (50 mM TrisCl pH 7.5, 100 mM NaCl, 8 mM MgSO_4_, 2.5 mM CaCl_2_). Aliquots were thawed on ice with intermittent vortexing to homogenize the sample. DNA was extracted using the ZymoBIOMICS DNA Miniprep Kit (Cat# D4300, ZymoResearch) and a Mini-BeadBeater 8 (Biospec Products, Bartlesville, OK) using 5 rounds of 1-minute homogenization followed by at least 1 minute at 4°C to cool the lysate between rounds. Eluted DNA was quantified using the Qubit 1x dsDNA HS Assay Kit and the Qubit 4 fluorometer (Invitrogen, Eugene, Oregon and Invitrogen, Singapore respectively). DNA was submitted for 16S metagenomic sequencing using the Zymo Quick-16S Plus Library Prep method and sequenced on the NextSeq 2000 platform (Seq Center, Pittsburgh, PA). Species distribution was visualized at the genus level by QIIME 2 view (https://view.qiime2.org/)[(23)](https://sciwheel.com/work/citation?ids=7223637&pre=&suf=&sa=0&dbf=0). Relative abundance based on total reads mapping to each genera was calculated using Excel (Microsoft Office Professional Plus 2019) and graphed using Graph Pad Prism version 9.4.1.

Phage lysate preparation for treatment

An *E. faecium* isolate from the patient (DVT1320) was used as the host for infection with Φ9184 and ΦHi3. All liquid incubation steps occurred at 37°C and 170 rpm. The host strain was grown overnight to stationary phase in tryptic soy broth (TSB) (Becton Dickinson & Co., BD 211825). The culture was then diluted 1:30 into fresh TSB and CaCl_2_ and MgCl_2_ were each added to a final concentration of 1 mM. Once the culture reached an OD­­_600_ between 0.15 – 0.25, previously prepared phage lysate was added at multiplicity of infection (MOI) of 0.1. Infected cultures were monitored periodically until the OD_600_ began to decline, signaling phage-mediated bacterial lysis. Cultures were pulled from the incubator and allowed to complete infection overnight at 4°C. The following day, the cultures were cleared with a 15-minute centrifugation at 4000g. The supernatant was filtered through a 22μM polystyrene filter (Corning). The resulting lysate was concentrated roughly 20x using cellulose 30,000 nominal molecular weight limit (NMWL) centrifugal filtration devices (Centricon Plus-70, Merck-Millipore Ltd). Concentrated lysate was then sterilized again by passage through a 22 μM syringe filter (Fisherbrand, cat# 09-720-004) and formulated at the desired titer in 1xPBS (Sigma-Aldrich, P5493) with 10mM MgSO_4_ (Sigma, cat# M7506­). USP71 sterility testing was performed (Accugen Laboratories, Addison, IL) and endotoxin concentration was measured by LAL assay (Thermo Scientific, Waltham, MA).

Phage spot titer assay

Lytic activity of Φ9184 and ΦHi3 on *E. faecium* clinical isolates was measured using the soft agar overlay method. Briefly, overnight cultures of each isolate were grown at 37°C in TSB. A 1mL aliquot was pelleted and resuspended in an equal volume of SM+ buffer. Resuspended cultures were diluted 1:50 in 5 mL molten TSB top agar (0.35% Agar, 10mM MgSO_4_) and layered on top of TSB bottom agar (1.5% Agar, 20mM MgSO_4_) and allowed to solidify. 10-fold serial dilutions of phage lysates were made in SM+ buffer, and 5 μL of each dilution was spotted onto the top agar. Spots were allowed to dry, and then plates were incubated upright for 24 – 48h at 37°C. The least diluted sample with countable plaques was recorded and titers were calculated as plaque forming units (PFU) per mL of lysate. Efficiency of plating (EOP) was calculated by dividing the phage titer on the clinical isolate by the titer on the host used for phage production.

Serum neutralization assay

Serum neutralization assays were performed similarly as the spot titer assays above except that 20 μL of each phage lysate or SM+ buffer was added to 180 μL of patient sera and the mixture was incubated for 24 hours at 37°C. The pre-incubated lysates were then serially 10-fold diluted with SM+ buffer and spotted onto top agar as above. EOP was calculated by dividing the titer of phage incubated with serum by the titer of phage incubated with SM+ buffer alone. The same protocol was used to measure the non-specific inhibitory effects of human serum on phage activity using commercially available pooled normal human serum (CompTech, Texas).

ELISA

Ultra-purified phage lysates were prepared using previously published methods of cesium chloride gradient centrifugation [(24)](https://sciwheel.com/work/citation?ids=15750613&pre=&suf=&sa=0&dbf=0). A phage-specific ELISA protocol was adapted from Dedrick et al, 2021 [(15)](https://sciwheel.com/work/citation?ids=11345538&pre=&suf=&sa=0&dbf=0) and is summarized here. 96-well EIA microplates (Corning, CLS3590) were coated with either 100μL of coating buffer (carbonate-bicarbonate pH 9.6 Sigma-Aldrich, cat# C3041) as a no-phage control or 1 x 10^9^ PFU/mL of ultra-purified phage lysates diluted into coating buffer. Plates were sealed and incubated at 4°C for 20 – 24h. Wells were washed 5 times with 250μL PBST (PBS – Sigma-Aldrich, P5493; 0.05% Tween-20 – EMD Millipore Corp., 655205), then blocked for 20 – 24h in blocking buffer (PBST + 3% milk – Research Products International, M17200). Patient serum samples from observation day 181 (before Φ) and day 337 (when complete phage neutralization was first noted) were heat inactivated at 56°C for 30 mins, aliquoted and stored at -20°C until use. Starting at an initial dilution of 1:100, Serum samples were serially diluted 1:4 in blocking buffer. Block was removed from wells without washing, and 100μL of patient serum dilutions were added to each well in technical duplicate. Plates were sealed and incubated at 4°C for 20 – 24h. Wells were then washed 5 times with 250μL PBST. HRP-conjugated secondary antibody (goat anti-human IgG Fc, Abcam, Cat# ab98624) was diluted 1:10,000 in PBST and 100μL was added to each well. Plates were sealed and incubated in the dark at room temperature for 1h. Wells were washed 2x with 250μL PBST followed by 3x 250μL PBS. 100μL of HRP substrate (3,3’,5,5’-tetramethylbenzidine, Sigma, cat# T0440) was added to each well and incubated in the dark at room temperature for 8 mins. The reaction was then stopped by adding 100μL 2N H_2_SO_4_ and absorbance at 450 nm was measured on a BioTek Synergy H1 plate reader (BioTek, Winooski, VT). This experiment was performed in biological triplicate.

Background absorbance of the plate and regents was calculated as the average absorbance of control wells without phage or serum added, and this was subtracted from the absorbance of each experimental condition. In order to compensate for any non-specific binding of the patient’s sera to the plate, the average absorbance of serum from day 181 and day 337 in no phage added control wells were subtracted from the corresponding absorbance of each serum condition in both Φ9184 and ΦHi3 coated experimental wells. All replicate values were used to fit an overall curve using a 4-parameter sigmoidal function linear regression as shown in Fig. 2C and calculate individual -log_10_EC50s for each replicate. The distribution of IgG -logEC50s of serum collected on day 181 versus day 337 were compared using a two-tailed t-test for both Φ9184 and ΦHi3.

*In vitro* phage resistance assay

All phage resistance experiments were performed in Todd-Hewitt media (Becton Dickinson & Co., BD 249240) supplemented with 10mM MgSO_4_. A top agar phage suspension was generated by adding 1 x 10^8^ PFU of either Φ9184 or ΦHi3 to molten top agar (0.35% agar) and plated onto warmed bottom agar plates (1.5% agar) and allowed to solidify. Negative control suspensions contained only top agar. The phage host strain DVT1320 was grown to late-log phase and then serially diluted 1:10 in sterile 1x PBS (Sigma-Aldrich, P5493) across 11 dilution steps. Five microliters of each dilution step were then spotted onto the solidified phage suspension, allowed to dry and then incubated overnight at 37°C. The following day, colony forming units (CFUs) were counted at dilution steps that allowed for accurate numeration (1 – 20 CFUs per spot). The negative control plates were used to calculate the estimated total CFUs present in each dilution step without phage exposure. For each phage suspension plate, the number of countable CFUs was divided by the total CFUs as estimated by the negative control calculations. This yielded a “resistance rate” or the estimated number of CFUs that would contain at least 1 phage-resistant mutant. This experiment was performed in biological triplicate.

Cross streak assays based on [(25)](https://sciwheel.com/work/citation?ids=2922738&pre=&suf=&sa=0&dbf=0) were used to confirm that the colonies isolated from the phage suspension plates were stably phage resistant. Briefly, >15 potentially resistant colonies were picked from the phage suspension plates and grown overnight in fresh media. The following day 1 x 10^8^ PFU of either Φ9184 or ΦHi3 was streaked across the center of a warmed bottom agar plate and allowed to dry. Ten microliter samples from each colony’s overnight culture or the phage host strain (DVT1320) were spotted onto the agar a few centimeters above the phage line. The plate was then tilted and the spots were allowed to run down the plate, crossing the phage line at a right angle. Plates were allowed to dry and then incubated overnight at 37°C. The following day, if the path of the culture sample was disrupted at the phage line and showed decreased cell density beyond it, then the colony was determined to be phage susceptible. However, if the path was not disrupted but rather had a uniform culture density across the entire path, it was determined to be phage resistant.

Phage-antibiotic growth suppression assay

BSI isolate 20, sampled from the patient’s final bacteremia event, was grown to stationary phase overnight in tryptic soy broth (Becton Dickinson & Co., BD 211825) supplemented with 10mM MgSO_4_ and 50 mg/L CaCl_2_. Bacteria were then diluted 1:100 into fresh media and allowed to enter log-phase before normalizing with additional fresh media to an OD to 0.1. Aliquots were then treated with either 0.5xMIC of daptomycin (Tocris Bioscience 39-171-0, FisherScientific); Φ9184, ΦHi3 or a 50:50 cocktail at an MOI of 0.05; or combinations of these. Plates were then incubated at 37°C with intermittent shaking in a BioTek plate reader (BioTek, Winooski, VT). and the OD600 was measured every 30 minutes. The assay was performed in biological duplicate.

Statistical analysis

All statistical analyses and graphing were performed in Graph Pad Prism version 9.4.1. Replicate values were summarized with mean values and standard error means. Two-tailed t-tests were used to compare between conditions (unless otherwise specified in the text or figure legend) and p-values <0.05 were considered statistically significant.

Data Availability

Genome sequencing data for all isolates was submitted to NCBI under BioProject PRJNA901969, with accession numbers listed in Table S1.

**References**

[17.   Wick RR, Judd LM, Gorrie CL, Holt KE. 2017. Unicycler: Resolving bacterial genome assemblies from short and long sequencing reads. PLoS Comput Biol 13:e1005595.](https://sciwheel.com/work/bibliography/3964715)

[18.   Stamatakis A. 2014. RAxML version 8: a tool for phylogenetic analysis and post-analysis of large phylogenies. Bioinformatics 30:1312–1313.](https://sciwheel.com/work/bibliography/265260)

[19.   Howden BP, Holt KE, Lam MMC, Seemann T, Ballard S, Coombs GW, Tong SYC, Grayson ML, Johnson PDR, Stinear TP. 2013. Genomic insights to control the emergence of vancomycin-resistant enterococci. MBio 4:e00412-13.](https://sciwheel.com/work/bibliography/3974047)

[20.   Duchêne S, Holt KE, Weill F-X, Le Hello S, Hawkey J, Edwards DJ, Fourment M, Holmes EC. 2016. Genome-scale rates of evolutionary change in bacteria. Microb Genom 2:e000094.](https://sciwheel.com/work/bibliography/5151535)

[21.   Chen S, Zhou Y, Chen Y, Gu J. 2018. fastp: an ultra-fast all-in-one FASTQ preprocessor. Bioinformatics 34:i884–i890.](https://sciwheel.com/work/bibliography/5861897)

[22.   Wood DE, Lu J, Langmead B. 2019. Improved metagenomic analysis with Kraken 2. Genome Biol 20:257.](https://sciwheel.com/work/bibliography/8120680)

[23.   Bolyen E, Rideout JR, Dillon MR, Bokulich NA, Abnet CC, Al-Ghalith GA, Alexander H, Alm EJ, Arumugam M, Asnicar F, Bai Y, Bisanz JE, Bittinger K, Brejnrod A, Brislawn CJ, Brown CT, Callahan BJ, Caraballo-Rodríguez AM, Chase J, Cope EK, Da Silva R, Diener C, Dorrestein PC, Douglas GM, Durall DM, Duvallet C, Edwardson CF, Ernst M, Estaki M, Fouquier J, Gauglitz JM, Gibbons SM, Gibson DL, Gonzalez A, Gorlick K, Guo J, Hillmann B, Holmes S, Holste H, Huttenhower C, Huttley GA, Janssen S, Jarmusch AK, Jiang L, Kaehler BD, Kang KB, Keefe CR, Keim P, Kelley ST, Knights D, Koester I, Kosciolek T, Kreps J, Langille MGI, Lee J, Ley R, Liu Y-X, Loftfield E, Lozupone C, Maher M, Marotz C, Martin BD, McDonald D, McIver LJ, Melnik AV, Metcalf JL, Morgan SC, Morton JT, Naimey AT, Navas-Molina JA, Nothias LF, Orchanian SB, Pearson T, Peoples SL, Petras D, Preuss ML, Pruesse E, Rasmussen LB, Rivers A, Robeson MS, Rosenthal P, Segata N, Shaffer M, Shiffer A, Sinha R, Song SJ, Spear JR, Swafford AD, Thompson LR, Torres PJ, Trinh P, Tripathi A, Turnbaugh PJ, Ul-Hasan S, van der Hooft JJJ, Vargas F, Vázquez-Baeza Y, Vogtmann E, Caporaso JG. 2019. Reproducible, interactive, scalable and extensible microbiome data science using QIIME 2. Nat Biotechnol 37:852–857.](https://sciwheel.com/work/bibliography/7223637)

[24.   Sambrook J, Fritsch EF, Maniatis T. 1989. Bacteriophageλ vectors, p. 2.1–2.125. *In* Nolan, C (ed.), Molecular cloning: a laboratory manual, 2nd ed. Cold Spring Harbor Laboratory Press, Cold Spring Harbor, N.Y.](https://sciwheel.com/work/bibliography/15750613)

[25.   Duerkop BA, Huo W, Bhardwaj P, Palmer KL, Hooper LV. 2016. Molecular basis for lytic bacteriophage resistance in enterococci. MBio 7(4):e01304-16.](https://sciwheel.com/work/bibliography/2922738)


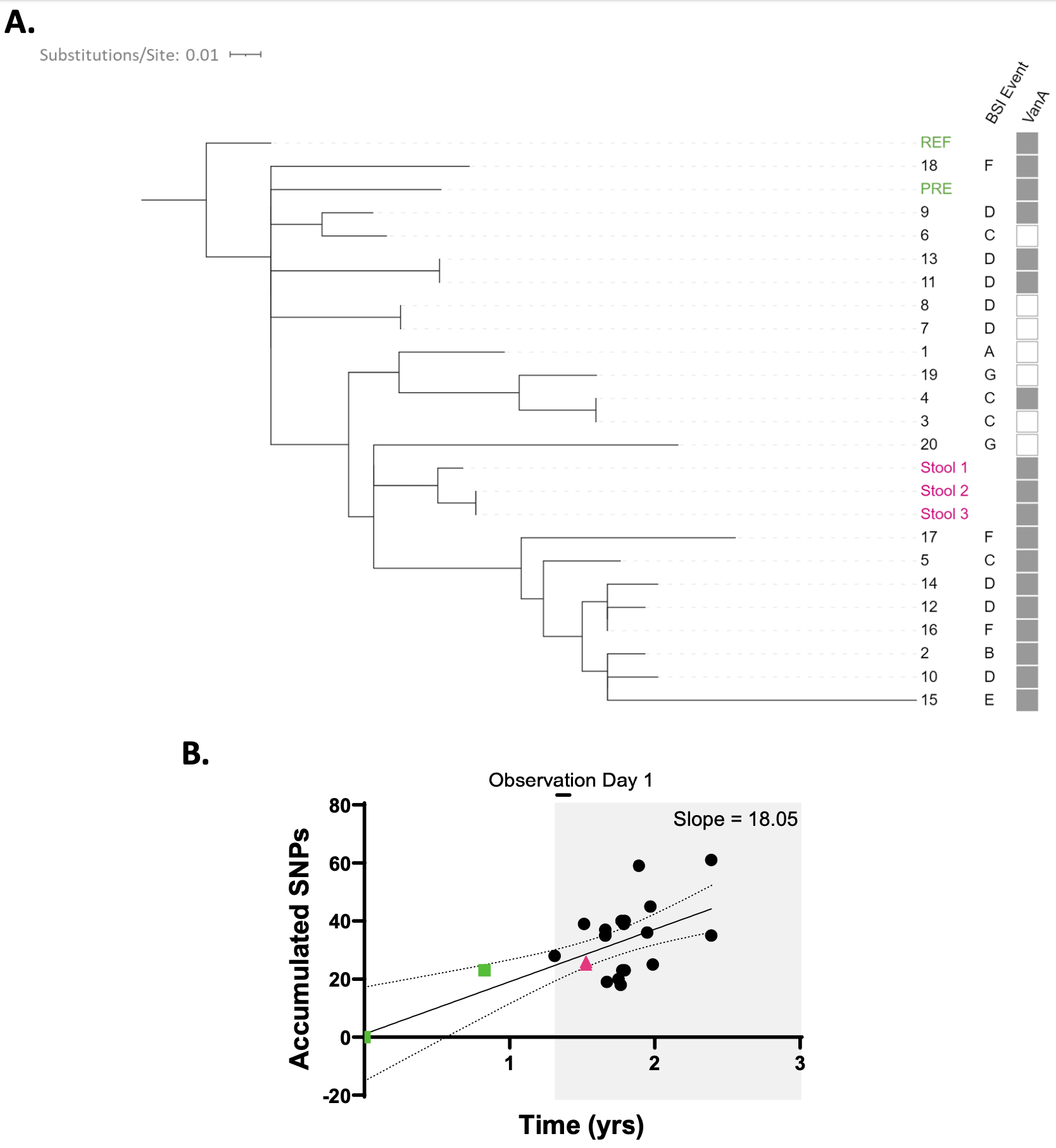


**Supplemental Figure 1. Genomic analysis of recurrent *E. faecium* BSI isolates.** (A) Core genome SNP-based maximum likelihood phylogenetic tree built with RAxML with 100 bootstraps. BSI isolate IDs are listed to the right of each leaf. Pre-observation isolates are labeled in green. The earliest pre-observation isolate (collected 18 months before the observation period) served as the reference genome (REF) and another pre-treatment isolate (PRE) was collected 6 months prior to the observation period. Invasive BSI isolates are numbered in black, in the order of isolation (see Fig. 1A for clinical timeline) and stool sample isolates are labeled in pink (Stool). The corresponding BSI events are listed (A – G, see Fig 1A). Right-most color strip indicates presence (grey) or absence (white) of the VanA operon. (B) Accumulated core genome SNPs over time compared to the reference genome (at origin). Pre-observation isolates (REF and PRE) are green squares, invasive BSI isolates are depicted by black circles and the three stool isolates are depicted by pink triangles. The best fit line estimates the overall SNP accumulation rate of 18 SNPs/year. Dotted curves indicate the 95% confidence bands. The gray background shading indicates the observation period (June 2020 – July 2021).


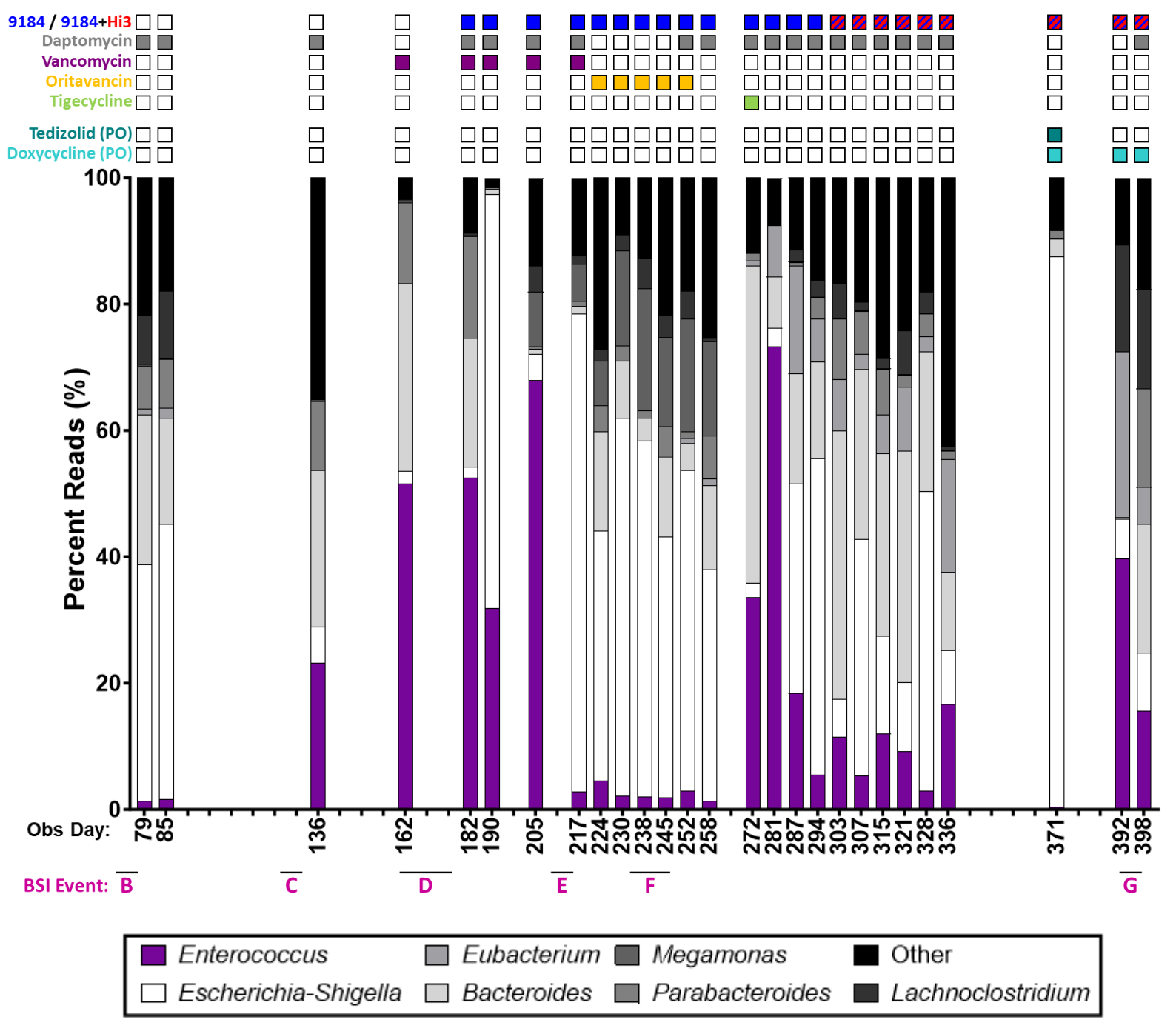


**Supplemental Figure 2. Stool 16S sequencing data.** 16S rRNA sequencing of the same stool samples analyzed in Figure 1B. Bar graph shows the relative abundance of reads mapping to the 7 most abundant genera, which included *Enterococcus* (purple). The other 6 most abundant genera are in grayscale and the percentages of reads mapping to all other genera are in black. Colored squares above each bar indicate which phage (top row), systemic intravenous antibiotics (rows 2 – 5) and oral antibiotics (rows 6 – 7) the patient was being treated with at the time each stool sample was collected. Numbers and horizontal lines below the bar graph highlight the day of observation and BSI events originally depicted in Figure 1A.


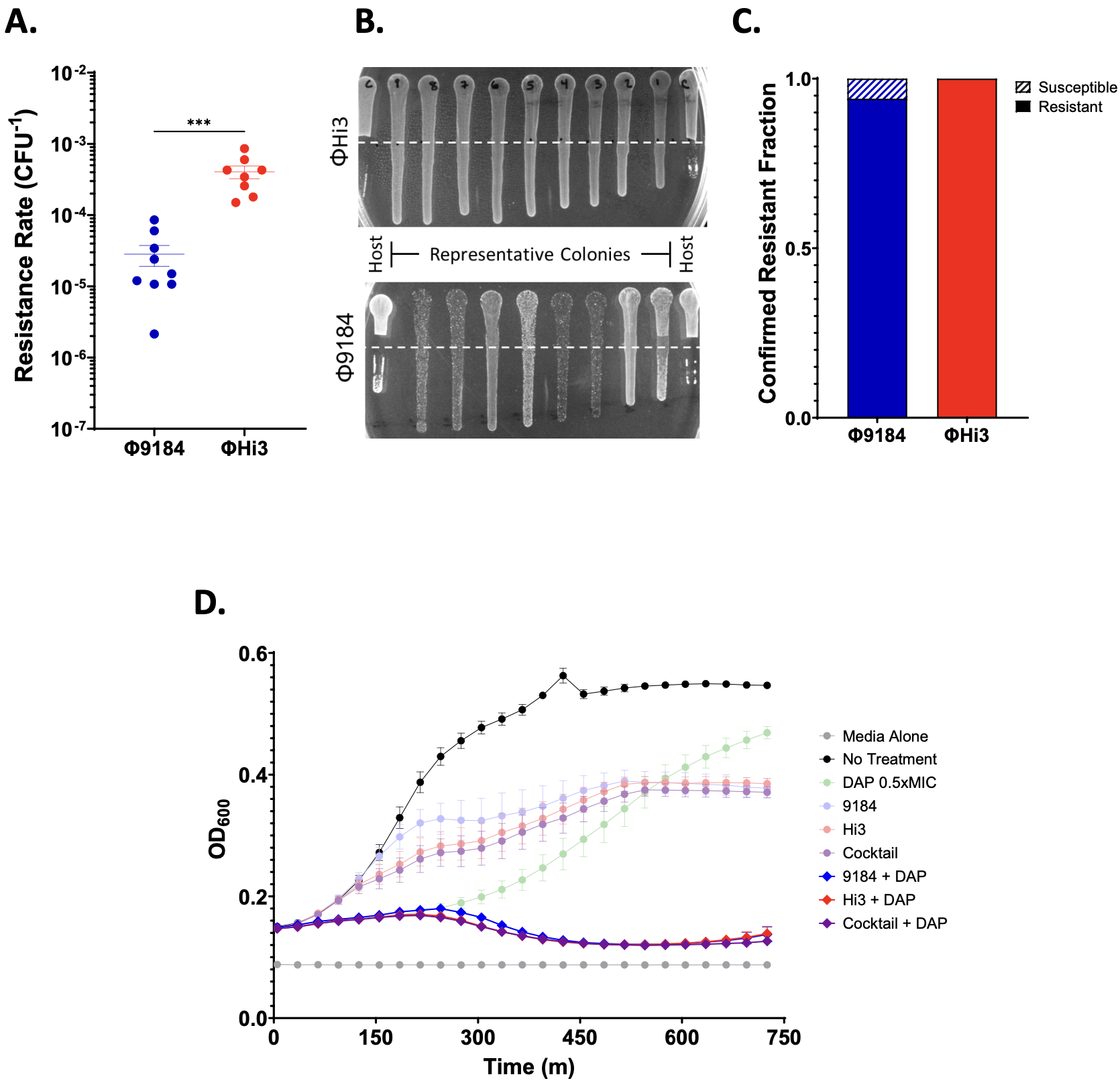


**Supplemental Figure 3. *In vitro* phage resistance quantification and enhanced growth suppression by daptomycin-phage combinations against the final breakthrough BSI isolate.** (A) Scatter plot of the *in vitro* rate of resistance for Φ9184 (blue) and ΦHi3 (red). Error bars depict the SEM of biological replicates. Rates of resistance are depicted as 1/minimum CFU input required to isolate a single phage resistant colony. (B) Images of phage cross streaks with representative phage-resistant mutants. Phage sensitive controls were the host strain. A phage-sensitive isolate will have a break in the bacterial track as it crosses the horizontally applied phage lysate (white dotted line), as seen in the host control lanes. Bacterial tracks of resistant clones are unbroken. (C) Quantification of colonies isolated in the *in vitro* resistance assay in panel A that were confirmed to be phage-resistant in panel B. (D) Bacterial isolate 20, from the final BSI event G, was treated with either 2µg/mL daptomycin (0.5xMIC in the experimental media); Φ9184, ΦHi3 or a 50:50 cocktail of both phages at a MOI of 0.05; or combinations of phage and daptomycin. The OD_600_ was measured every 30 minutes, and the graph depicts the average and SEM of the OD_600_ over a 12-hour period for each experimental condition as compared to a growth control (No Treatment). Wells containing media alone indicate the background absorbance of the assay. Combinations of daptomycin and phage were the most effective at suppressing bacterial growth of the final breakthrough isolate, suggesting that breakthrough BSIs were unlikely due to phage-antibiotic antagonism.


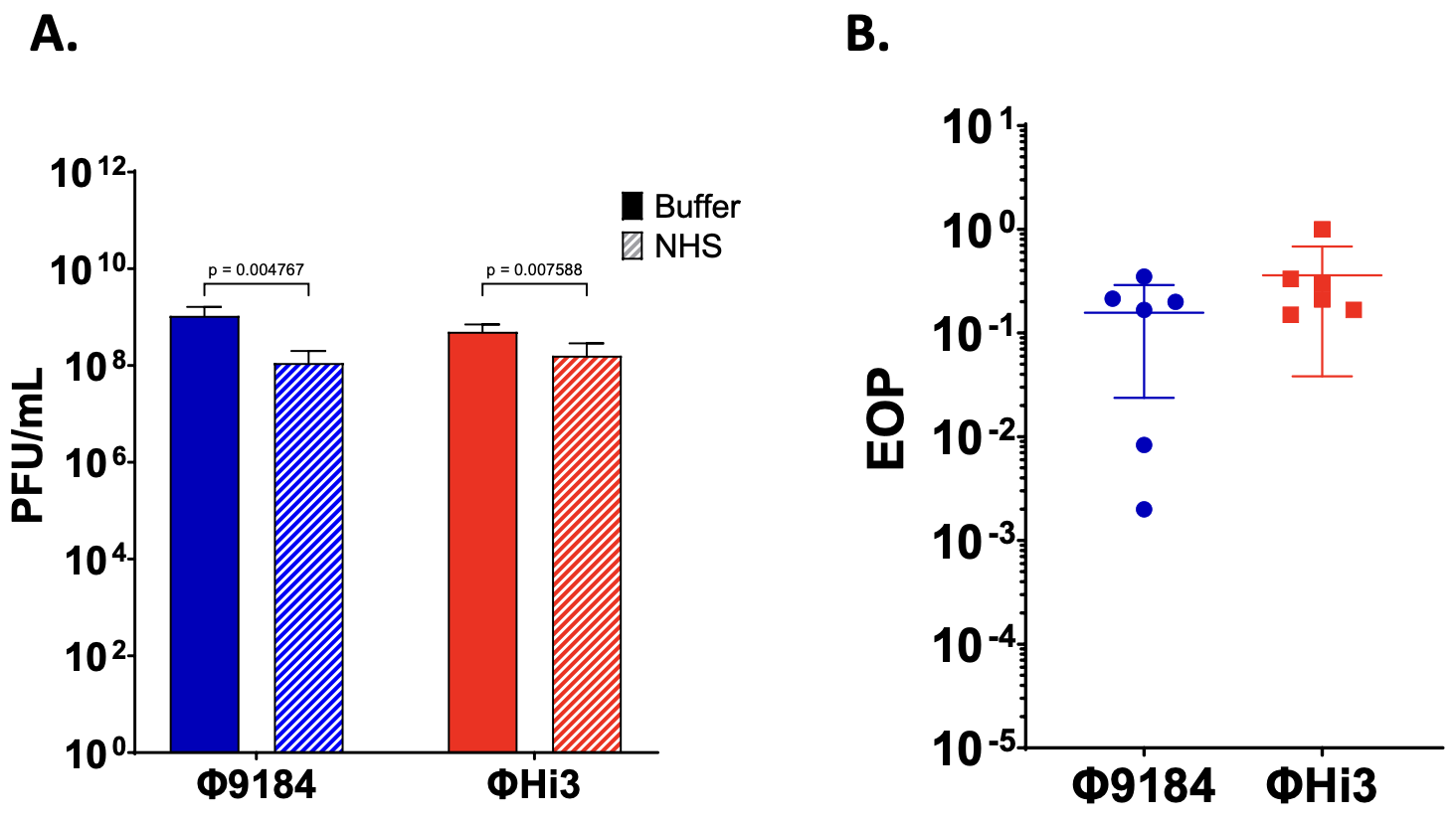


**Supplemental Figure 4. Pooled normal human serum control for serum neutralization experiments.** (A) The average titer and SEM of Φ9184 or ΦHi3 on the host strain after a 24-hour incubation with either SM+ phage buffer (Buffer) or commercially available pooled normal human serum (NHS). Reported p-values are from an unpaired t-test. (B) The same data is also depicted as an efficiency of plating (EOP) of each replicate, which compares the titers of each phage after serum incubation to that of the buffer control. Error bars depict the SEM. NHS control experiments indicate that human serum alone can modestly and non-specifically neutralize phage activity.
